# Metal-Chelated Polymeric Nanomaterials for the Removal of Penicillin G Contamination

**DOI:** 10.3390/polym15132832

**Published:** 2023-06-27

**Authors:** Cansu İlke Kuru, Fulden Ulucan-Karnak, Sinan Akgol

**Affiliations:** 1 Department of Biochemistry, Faculty of Science, Ege University, 35100 Izmir, Turkey; cansuilke89@gmail.com (C.İ.K.); sinan.akgol@ege.edu.tr (S.A.); 2 Biotechnology Department, Graduate School of Natural and Applied Sciences, Ege University, 35100 Izmir, Turkey; 3 Advanced Biomedical Technologies Department, Graduate School of Natural and Applied Sciences, Ege University, 35100 Izmir, Turkey

**Keywords:** nanoparticles, β-lactam antibiotics, penicillin G, removal

## Abstract

We developed selective and relatively low-cost metal-chelated nanoparticle systems for the removal of the penicillin G (Pen G) antibiotic, presented for the first time in the literature. In the nanosystem, poly(glycidyl methacrylate) nanoparticles were synthesized by a surfactant-free emulsion polymerization method and covalently bound with a tridentate-chelating ligand, iminodiacetic acid, based on the immobilized metal chelate affinity technique. It was modified with Cu^2+^, a chelating metal, to make Pen G specific. Metal-chelated nanoparticles were characterized by Fourier-transform infrared spectroscopy, energy dispersive spectrometry, zeta dimensional analysis, and scanning electron microscopy technology. Optimization studies of the Pen G removal were conducted. As a result of this study, Pen G removal with the p(GMA)-IDA-Cu^2+^ nanoparticle reached its maximum adsorption capacity of 633.92 mg/g in the short time of 15 min. The Pen G adsorption of p(GMA)-IDA-Cu^2+^ was three times more than that of the p(GMA) nanoparticles and two times more than that of the ampicillin adsorption. In addition, there was no significant decrease in the adsorption capacity of Pen G resulting from the repeated adsorption–desorption process of metal-chelated nanoparticles over five cycles. The metal-chelated nanoparticle had an 84.5% ability to regain its ability to regenerate the product with its regeneration capability, making the widespread use of the system very convenient in terms of reducing cost, an important factor in removal processes.

## 1. Introduction

Beta-lactam antibiotics are the most important group of antibiotics drugs, and they have been used as antimicrobial drugs for almost 80 years. The use of beta-lactam antibiotics as a treatment to infections has attracted considerable interest in recent years [1]. Antibiotics are released into the environment through end-use wastewater as a result of their use as human and animal treatments, as well as many field growth factors, such as animal breeding and fish farming [2,3,4,5]. In addition, one of the main sources of antibiotic contamination in the environment is the involvement of sewage sludge in plants and soil [6]. The presence of antibiotic residues in animal products and environmental wastewater has been legislated by the Council of Europe (Council Directive 96/23/EU), and the Food and Drug Administration (FDA) requires monitoring of the levels determined for countries’ safety assessments [7]. These antimicrobials are on the list of priority and dangerous substances of the European Union Water Quality, Composition Directives [6,7,8,9]. Recently, antibiotic and bacterial resistances have emerged in Europe and North America because of the contamination of antibiotic wastes with ng/L–μg/L concentrations from the waste of pharmaceutical companies and hospitals wastewater to surface or groundwater [10]. Penicillins are important beta-lactam antibiotics frequently used for infectious diseases in humans. In veterinary medicine, antibiotics are used for curative and preventive treatment of breast inflammation and respiratory and enteric diseases. These compounds are also used as growth-promoting agents in livestock as a food additive. Penicillin G, a common antibiotic, is a penicillin derivative used in the treatment of diseases in the form of sodium and potassium salts and the group of beta-lactam antibiotics. It is mostly used against Gram-positive bacteria and the Gram-negative strain-related variety of infectious disorders. Penicillin G is an antibiotic that dissolves in water and works by stopping the formation of peptidoglycan to break down the cell walls of bacteria [11]. A portion of Pen G may pass through the aerobic and anaerobic treatment units of wastewater treatment facilities. Antibiotics must, therefore, approach a permissible discharge limit before being disposed of into the environment and aquatic ecosystems due to the health risk associated with their release into the environment [12].

An important risk for such widespread use of antibiotics is the development of antibiotic-resistant bacterial strains. These strains can be a significant threat to public health, and there is a concern that the unlimited use of antibiotics will endanger our ability to fight infections in the future [13,14]. Another problem associated with the presence of antibiotic residues in meat and dairy products is the risk of an allergic reaction in hypersensitive individuals and the induction of production-initiating cultures in the dairy industry [15,16]. Since water quality is one of the most important issues concerning environmental chemistry and human health, the prospect of removing antibiotic residues from wastewater increases in importance [17].

Microbial inhibition tests, enzymatic tests, enzyme-linked immunoassays, and chromatographic techniques are used for antibiotic detection from wastewater and food products. However, the methods developed are ineffective and not selective due to the stable nature of the antibiotics and their extremely low biodegradability. Traditional biochemical techniques used for the use of Pen G wastes in water or food products are expensive and require complex devices and materials [18]. For this reason, there is a significant increase in the demand for reliable and sensitive analytical methods for antibiotic analysis. In recent times, nanoparticles have been used in many biotechnological applications due to their physical and chemical properties. Nanoparticles synthesized according to different shapes and functions have been used in purification, separation of cells, protein and enzyme immobilization, clinical diagnosis, and imaging. The use of nanoparticles is also recommended for the removal of antibiotics that are released into the environment. The adsorption capacities of nanoparticles are high because of their large predominant surface area [19,20,21].

In this study, we developed a selective and relatively low-cost methacrylate-based nanoparticle system for the removal of penicillin G antibiotics, presented for the first time in the literature. Firstly, metal-chelated nanoparticles, p(GMA), were prepared by the surfactant-free emulsion polymerization method. After that, nanoparticles were modified with iminodiacetic acid (IDA) and Cu. Fourier-transform infrared spectroscopy (FTIR), elemental analysis, zeta size analysis, and scanning electron microscopy (SEM) technologies were used for the characterization of the nanoparticles. The optimization of the Penicillin G (Pen G) removal with p(GMA)-IDA-Cu^2+^ nanoparticles was investigated at different conditions. The p(GMA)-IDA-Cu^2+^ nanoparticles were developed for the removal of antibiotics, and they can be used easily in the food and chemical industries in large-scale production.

## 2. Materials and Methods

### 2.1. Materials

Pen G sodium salt, glycidyl methacrylate (GMA), methanol (MeOH), and other chemicals, such as potassium persulfate (KPS), polyvinyl alcohol (PVA), ethylene glycol dimethacrylate (EGDMA), imino diacetic acid (IDA), CuSO_4_, were purchased from Sigma-Aldrich company.

### 2.2. Synthesis of p(GMA)-IDA-Cu^2+^ Nanoparticles

The preparation of the p(GMA) nanoparticle was carried out as follows: 0.275 g PVA was dissolved by heating with 25 mL distilled water, and 0.6 mL GMA and 0.3 mL EGDMA were prepared and added to the PVA. This solution medium was purged with nitrogen for 5 min. The medium was finally added with 0.0198 g of initiator KPS solution dissolved in 45 mL of water with agitation and mixed slowly. The prepared polymerization medium was sealed, and the temperature was gradually increased to 70 °C by shaking in a water bath. When milk had a white color, a p(GMA) nanoparticle was prepared.

Following the nanoparticle preparation step, precipitation, washing, and preparation were performed. For washing, the centrifuge tube was centrifuged at 30,000× *g* for 45 min, washed with 70 mL of purified water, and then centrifuged again at 30,000× *g* for 45 min. The same procedure was repeated with ethanol, and then the nanoparticle was sonicated with 70 mL of water. The prepared p(GMA) nanoparticle was stored at +4 °C until use [22].

To modify the p(GMA) nanoparticles with the chelating agent IDA, 0.8 g of IDA and 50 mL of 2 M Na_2_CO_3_ solution were added over 0.05 g p(GMA) nanoparticles. The pH of the medium was set to 11. The mixture was then allowed to stand for 18 h at 70 °C. Nanoparticles were centrifuged at 30,000× *g* for 45 min, washed with 70 mL of distilled water, and centrifuged again at 30,000× *g* for 45 min. The nanoparticles were sonicated in 50 mL of distilled water. The prepared p(GMA)-IDA nanoparticles were stored at +4 °C until use [23].

**Figure 1:**
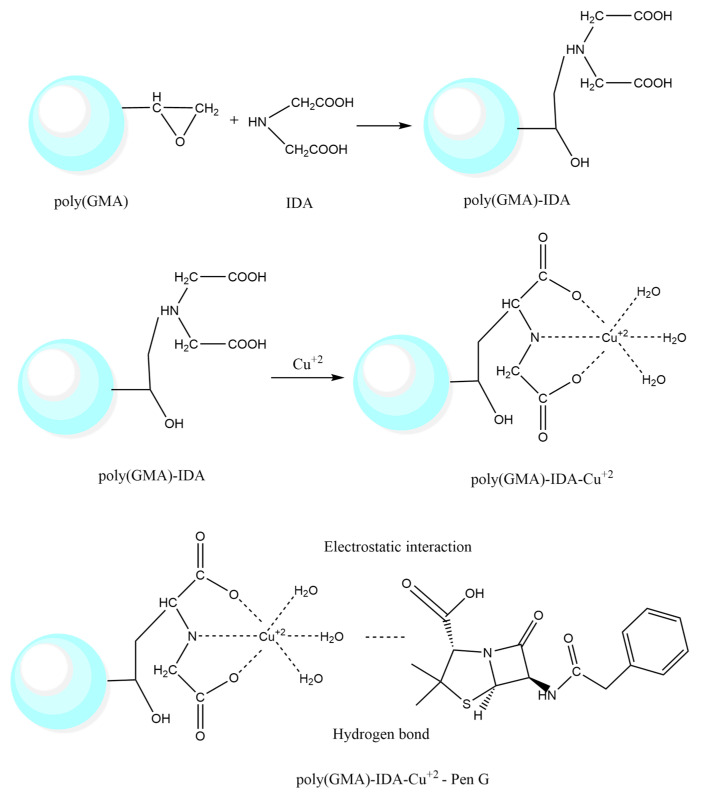
Schematic representation of p(GMA)-IDA-Cu^2+^ and p(GMA)-IDA-Cu-Pen G nanoparticles.

For binding of Cu^2+^ to p(GMA)-IDA structures, 45 mL p(GMA)-IDA nanoparticles were precipitated by centrifugation at 30,000× *g* for 45 min, and approximately 1 g nanoparticle was added to 30 ppm 50 mL pH 4.1 CuSO_4_ solution. The prepared solution was allowed to stir at room temperature for 2 h. At the end of the period time, the nanoparticles were centrifuged at 30,000× *g* for 45 min, centrifuged again at 30,000× *g* for 45 min after washing with 70 mL of purified water, and the nanoparticles were sonicated by dispersing 50 mL of water. The prepared Cu-bound p(GMA)-IDA nanoparticles were stored at +4 °C until use [24]. Schematic representations p(GMA)-IDA-Cu and p(GMA)-IDA-Cu-Pen G nanoparticles are shown in Figure 1.

### 2.3. Characterization of p(GMA)-IDA-Cu^2+^ Nanoparticles

Spherical morphology and size of the p(GMA)-IDA-Cu^2+^ nanoparticles were determined by SEM technology. SEM images of p(GMA) and p(GMA)-IDA-Cu^2+^ nanoparticles were obtained by scanning electron microscopy (SEM) (JEOL JSM-7600F). The morphological characteristics and size of the particle were studied. The atomic percentages of the elements in the p(GMA)-IDA-Cu^2+^ nanoparticles were measured using Energy Dispersive X-ray Spectroscopy (JEOL JSM-7600F).

Chemical structures and bonds were determined by FTIR-ATR technology. The FTIR-ATR spectrum of p(GMA), p(GMA)-IDA, p(GMA)-IDA-Cu^2+^, and p(GMA)-IDA- Cu^2+^-Pen G nanoparticles were obtained using FTIR-ATR spectrophotometer (Thermo FTIR). The dried nanoparticles (2 mg) were analyzed by obtaining a spectrum in the range of 4000–500 cm^−1^ wave number.

The zeta size analysis of the p(GMA), p(GMA)-IDA, and p(GMA)-IDA-Cu^2+^ nanoparticles were performed with a Malvern Zeta-sizer instrument. The nanoparticles were prepared to be very dilute and were delivered to the cell of the device. For this purpose, dimension measurement was performed by injecting a 1 mL dilute polymer solution into the sample well of the zeta dimension analyzer.

In addition, the dry mass of the nanoparticle was determined, the mass of dry polymer in mL was calculated in mg, and the specific surface area of the polymer was calculated using these data.

Equation (1) was used to determine the surface area of synthesized p(GMA)-IDA-Cu nanoparticles, giving the number of particles in the 1 mL suspension [25]:

N = 6 × 10^10^ × S/π × ρ_s_ × d^3^ (1)

where N is the number of nanoparticles in the 1 mL suspension; S is percent solids (%); d is the diameter (μm); and ρs is the polymer density (g/mL).

The amount of mg nanoparticle in the mL suspension was theoretically determined using the standard mass–volume plots of the nanoparticles. The specific surface area of the synthesized p(GMA)-IDA-Cu nanoparticles was calculated in m^2^/g using the equilibrium surface area equation:

Surface area of sphere = 4 × π × r^2^ (π, 3,14; r, nanoparticle radius (m)).

### 2.4. High-Performance Liquid Chromatography (HPLC) Analysis for Pen G Assays

Pen G assays were performed in a Shimadzu SPD-10AVP HPLC System, 150 × 4.6 mm, 5 μm, C18 column. Here, 55:45 (*v*:*v*) Methanol: 50 mM Phosphate buffer pH 3 was used as the mobile phase. The flow rate was set to 1.2 mL/min, and analysis at ambient temperature was performed. The UV detector used a wavelength of 210 nm for Pen G. The ampicillin used in the selectivity assay was determined at 220 nm. The method used by Javanbakht et al. [26] for HPLC analysis was modified. The calibration curve was prepared at 5–100 μg/mL. The amounts of Pen G adsorbed to the surface of the gram nanoparticle were calculated using Equation (2) over the determined substance concentrations. In the following equation, Q: is the amount of Pen G adsorbed per gram nanoparticle (mg), C_initial_: is the initial concentration of Pen G, and C_final_: is the concentration of unbinding Pen G.

(2) $$Q=\frac{\left(C_{initial}-C_{final}\right)(mg/L)\times V_{final}(mL)}{\mathrm{Dry}\mathrm{Particle}\mathrm{Mass}\left(mg\right)\times 10^{-3}}$$

### 2.5. Optimization Studies for Removal of Pen G with p(GMA)-IDA-Cu^2+^ Nanoparticles

Optimization studies for the removal of Pen G with p(GMA)-IDA-Cu^2+^ nanoparticles in terms of time, pH, temperature, ionic strength, and initial Pen G concentration were investigated. Pen G potassium salt (626.6 mg 1,000,000 IU) [27] was dissolved in 2 mL of buffered water to prepare a Pen G solution at a concentration of 313.3 mg/mL for use in adsorption experiments. Each buffer was 0.1 M for pH experiments; sodium citrate buffer for pH 4, and phosphate buffer were prepared for pH values of 5, 6.5, and 7.4. Adsorption capacity value (Q) was calculated by HPLC analysis after the experiments; g denotes the amount of Pen G adsorbed per polymer.

### 2.6. pH Effect on Pen G Removal

To investigate the pH effect on Pen G removal, 0.1 M concentration buffers prepared at different pH values were used. Experiments were performed at room temperature. Here, 100 μL of Pen G solution and 100 μL of polymer solution were added to 800 μL buffer solution to give a final volume of 1 mL; the initial Pen G concentration was 0.05 mg/mL, operated at 25 °C with an angled mixer. To investigate the pH effect of the unmodified p(GMA) nanoparticle, 100 μL of Pen G solution and 100 μL of polymer solution were added to 800 μL buffer, and the angled mixer was operated. After 2 h of adsorption, the samples were centrifuged at 14,500× *g* for 20 min, and Pen G analysis was performed on their upper phases. For each pH value, separate media were added in the same manner but with pure water instead of polymer, and this was used to determine the initial concentration in HPLC analysis.

### 2.7. Initial Concentration Effect on Pen G Removal

To investigate the effect of initial concentration, the initial Pen G concentration in the medium was adjusted to 800 μL 0.1 M pH 6.5 phosphate buffer as 0.05, 0.1, 0.5, 1, 2, and 4 mg/mL, and final volumes of 1 mL 100 μL of Pen G and 100 μL of polymer were added to the concentration. To investigate the effect of the initial concentration of the unmodified p(GMA) nanoparticle, 100 μL of Pen G solution and 100 μL of polymer solution were added to 800 μL of pH 6.5 phosphate buffer, and the 2 h angled mixer was operated. Experiments were performed at room temperature. After adsorption, the samples were centrifuged at 14,500× *g* for 20 min, and Pen G analysis was performed on their upper phases. Polymer-free media were also used to determine the initial concentration for each concentration value.

### 2.8. Ionic Strength Effect on Pen G Removal

To investigate the effect of ionic strength, we added 800 μL of NaCl (0.05, 0.1, 0.5, 1, and 2 M) solution in different concentrations, 100 μL Pen G on 0.1 M pH 6.5 phosphate buffer solution, and 100 μL of polymer solution and stirred at 25 °C for 2 h. To investigate the time effect of the unmodified p(GMA) nanoparticle, 100 μL of Pen G solution and 100 μL of polymer solution were added to 800 μL, 0.1 M pH 6.5 phosphate buffer, and the angled mixer was operated. Experiments were performed at room temperature. After adsorption, the samples were centrifuged at 14,500× *g* for 20 min, and Pen G analysis was performed on their upper phases. Thus, the ionic severity-dependent change of adsorption was studied. Unlike the media prepared to determine the initial concentration in HPLC analysis, pure water was added instead of the polymer.

### 2.9. Time Effect on Pen G Removal

To examine the time effect, 100 μL of Pen G solution and 100 μL of polymer solution were added to 800 μL, 0.1 M pH 6.5 phosphate buffer, and the angled mixer was operated at 25 °C. Then, 100 μL of Pen G solution and 100 μL of polymer solution were added to 800 μL, pH 7.5 phosphate buffer and the angled mixer was operated to examine the time effect of the unmodified p(GMA) nanoparticle. Experiments were performed at room temperature. Samples (1 mL) were taken at 0, 5, 15, 30, 45, 60, 90, and 120 min from the adsorption media with an initial Pen G concentration of 0.05 mg/mL and centrifuged at 14,500× *g* for 20 min, and Pen G test was performed in the upper phases. Thus, the time-dependent change in adsorption was studied. In the blind trial to determine the initial concentration in HPLC analysis, pure water was added instead of polymer.

### 2.10. Temperature Effect on Pen G Removal

The effects of temperature were studied at 4 °C, 25 °C, 37 °C, and 55 °C. Experiments were performed in a 0.1 M pH 6.5 phosphate buffer at a volume of 1 mL, with an initial Pen G concentration of 0.05 mg/mL. Then, 100 μL of Pen G solution and 100 μL of polymer solution were added to 800 μL of buffer solution and stirred for 2 h in media which were adjusted to adsorption temperature. To investigate the temperature effect of the unmodified p(GMA) nanoparticle, 100 μL of Pen G solution and 100 μL of polymer solution were added to 800 μL, pH 6.5 phosphate buffer and an angled mixer was operated for 2 h. After adsorption, the samples were centrifuged at 14,500× *g* for 20 min, and Pen G analysis was performed on their upper phases. Polymer-free media were also mixed in the water bath for 1 h at each temperature and used to determine the initial concentration.

### 2.11. Reusability Experiment of Metal-Chelated Nanoparticles

For re-usability experiments, a 50 mL desorption solution of 2 M NaCl solution was prepared in 0.1 M pH 4 citrate buffer. The initial concentration of Pen G was 0.05 mg/mL and 0.1 mM phosphate buffer pH 6.5. After 1 h of adsorption at room temperature, the nanoparticles were centrifuged at 14,500× *g* for 20 min and then precipitated. The Pen G test was performed in the upper phase. A 1 mL desorption solution was placed on the precipitated nanoparticle, and desorption was carried out on the angled stirrer for 1 h. The nanoparticle precipitated at 14,500× *g* was reacted with distilled water for 30 min for sterilization after eachadsorption–desorption step. After the regeneration, the second cycle was performed in the same manner by adding the Pen G solution. The same polymer adsorption–desorption cycling was repeated 5 times so that the reproducibility of the synthesized nanoparticle could be determined in this way. The desorption ratio was calculated using the following equation:

(3) $$Desorption\;ratio\left(\%\right)=\frac{release\;the\;amount\;of\;Pen\;G\;into\;the\;desorption\;medium}{adsorption\;amount\;of\;Pen\;G}\times 100$$

### 2.12. Selectivity Experiment of p(GMA)-IDA-Cu^2+^ Nanoparticles

For the investigation of the selectivity of p(GMA)-IDA-Cu^2+^ nanoparticles, the nanoparticle was treated with ampicillin, which is another β-lactam antibiotic whose structure is very similar to that of Penicillin G. Experimental conditions were selected as (C_Penicillin_ G: 0.05 mg/mL, phosphate buffer pH = 6.5, T: 25 °C, t_adsorption_: 1 h) and (C_ampicillin_: 0.05 mg/mL, phosphate buffer pH = 6.5, T: 25 °C, t_adsorption_: 1 h). For investigation of specificity of developed nanoparticles, Pen G adsorption of p(GMA)-IDA-Cu^2+^ nanoparticles were compared with p(GMA) nanoparticles. The selectivity and specificity of the nanopolymer were calculated using the following equations (Equations (4)–(6)):

Distribution coefficient:

K_d_ = [(C_i_ − C_f_)/C_f_] ∗ (V/m) (4)

Selectivity:

K_Ampicillin_ = K_d Penicillin G_/K_d Ampicillin_ (5)

Specificity:

Kd_p(GMA)_ = K_d Penicillin G_/K_d p(GMA)_ (6)

## 3. Results

### 3.1. Characterization of the Metal-Chelated Nanoparticles

#### 3.1.1. FTIR-ATR Spectrum of p(GMA), p(GMA)-IDA, p(GMA)-IDA-Cu^2+^, and p(GMA)-IDA-Cu-Pen G Nanoparticles

**Figure 2:**
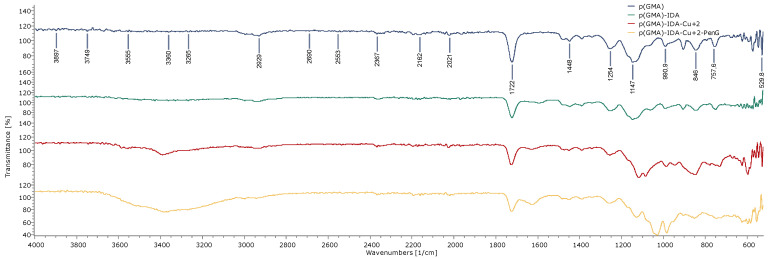
FTIR-ATR Spectrum of p(GMA) (**1**); p(GMA)-IDA (**2**); p(GMA)-IDA-Cu (**3**); p(GMA)-IDA-Cu-Pen G; and (**4**) Nanoparticles.

The FTIR-ATR spectrum of p(GMA), p(GMA)-IDA, p(GMA)-IDA-Cu^2+^, and p(GMA)-IDA-Cu-Pen G nanoparticles is shown in Figure 2. As seen from the FTIR-ATR spectrum, the peak at approximately 1147 cm^−1^ in the first spectrum of the p(GMA) nanoparticle was the band of epoxy groups in p(GMA). The reduction of the band intensity of the epoxy group in the seconf spectrum of the p(GMA)-IDA nanoparticle proves that the IDA functional group was linked to the p(GMA) ring-opening reaction, and the IDA molecule was linked through these groups. In addition, the peak of the carboxyl (-COOH) groups at 1722 cm^−1^ and the peak of the CN bond at 1448 cm^−1^ exhibited a dense peak in the B spectrum rather than the A spectrum, and this was due to the binding of the IDA molecule. In addition, in the third spectrum, the decrease in the same band intensity indicated that the Cu element was linked via carboxyl groups. The intensity of the band observed at approximately 3555 cm^−1^, which was related to the -OH group, increased in the spectrum C of the p(GMA)-IDA-Cu^2+^ nanoparticle concerning the second spectrum of p(GMA)–IDA. This shows Cu atoms were bound to p(GMA)-IDA. The peak due to -NH groups at 3360 cm^−1^ in the fourth spectrum of the p(GMA)-IDA-Cu-Pen G nanoparticle was due to the adsorption of Pen G [28,29].

#### 3.1.2. SEM Analysis of p(GMA)-IDA-Cu^2+^ Nanoparticles

**Figure 3:**
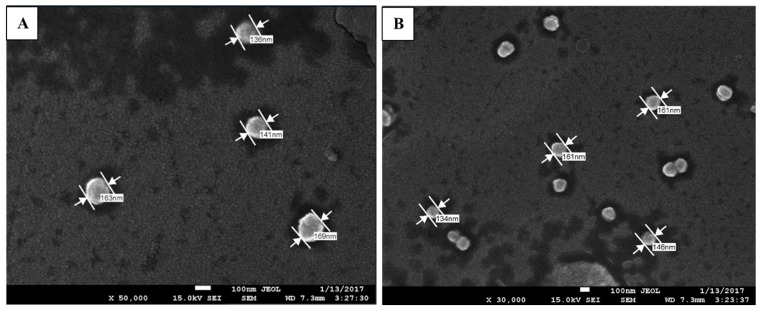
SEM Images of (**A**) p(GMA) and (**B**) p(GMA)-IDA-Cu^2+^ nanoparticles.

The morphological structure of the p(GMA) and p(GMA)-IDA-Cu^2+^ nanoparticles were observed in scanning electron microscopy images, as shown in Figure 3. It was observed that the particles were spherical, and their dimensions were approximately 150 nm for p(GMA)-IDA-Cu^2+^.

#### 3.1.3. Elemental Analysis of p(GMA)-IDA-Cu^2+^ Nanoparticles with EDS

**Figure 4:**
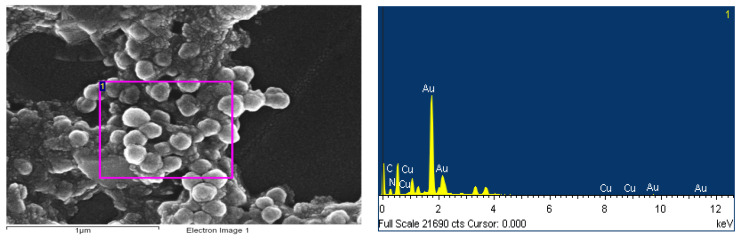
Results of elemental analysis for p(GMA)-IDA-Cu^2+^ nanoparticles with EDS.

The atomic percentages of the elements in the p(GMA)-IDA-Cu^2+^ nanoparticle was determined. As a result of the analysis conducted on the area of 1 μm^2^ from the surface of the nanoparticle, it was proved that the incorporation of IDA into the structure was accomplished by determining the presence of atom N 32.82% (Figure 4). The purple box shows the area of EDS analysis performed. 

#### 3.1.4. Zeta Size Analysis of p(GMA) Nanoparticles

**Figure 5:**
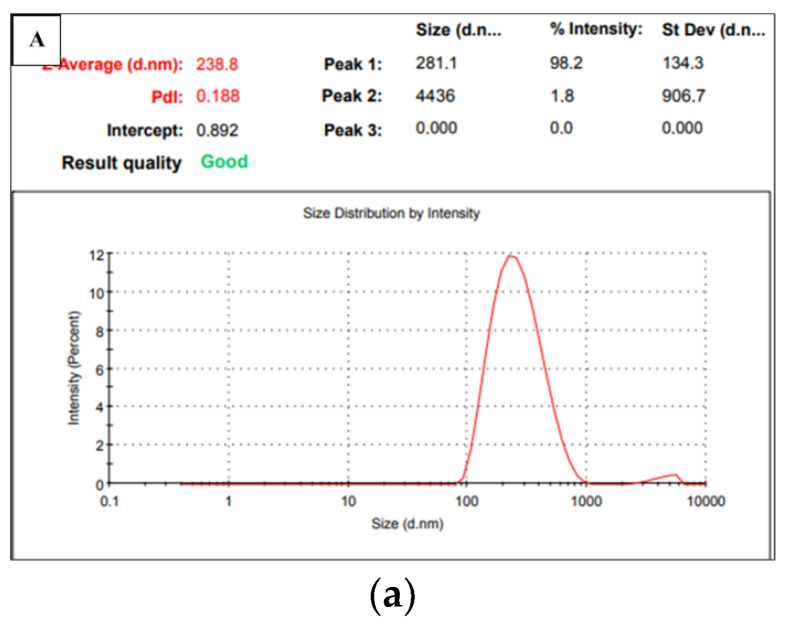

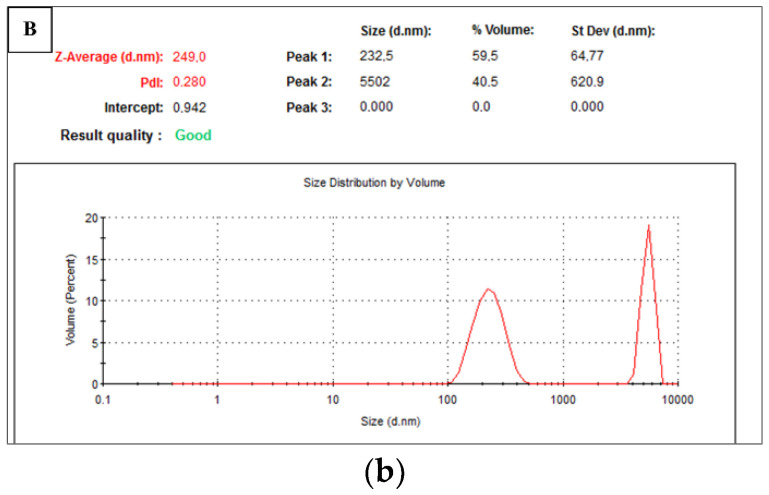

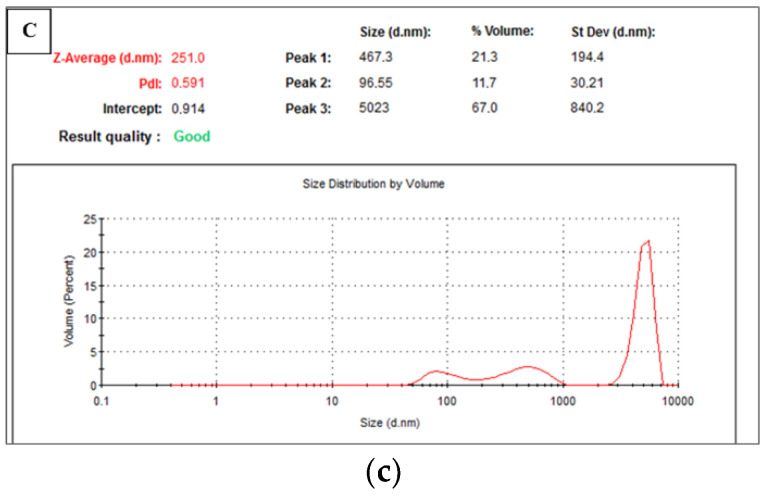
Zeta size analysis of (**A**) p(GMA); (**B**) p(GMA)-IDA; and (**C**) p(GMA)-IDA-Cu^2+^ nanoparticles.

For the zeta size analysis of the p(GMA), p(GMA)-IDA, and p(GMA)-IDA-Cu^2+^ nanoparticles, according to the results shown in Figure 5, the average particle size of the nanoparticles was determined to be 238.8, 249, and 251 nm, respectively. The polydispersity index was recorded as low as 0.188, 0.280, and 0.591. It was understood that the particle size distribution was in a narrow range. In a zeta size analysis, the size measurement is performed by utilizing the Brownian motion, which is the ability of the particles to move the liquid to determine the particle size. In this movement, small particles are fast, and large particles move slowly. During the measurement, dynamic light is sent by the device to the sample environment to produce information about the size of particle motion from its size [30]. It is also thought that these nanoparticles, which can be well dispersed in an aqueous medium, may cause the hydrophilic character to take up some water and swell the structure. It was, therefore, assessed that these situations caused the particle sizes to be larger than the actual size.

#### 3.1.5. Surface Area of p(GMA)-IDA-Cu^2+^ Nanoparticles

The diameters of p(GMA)-IDA-Cu nanoparticles were determined to be 150 nm, and the specific surface area was calculated as 1072.013 m^2^/g using Equation (1).

### 3.2. Removal of Pen G with Metal-Chelated Nanoparticles

#### 3.2.1. The pH Effect

**Figure 6:**
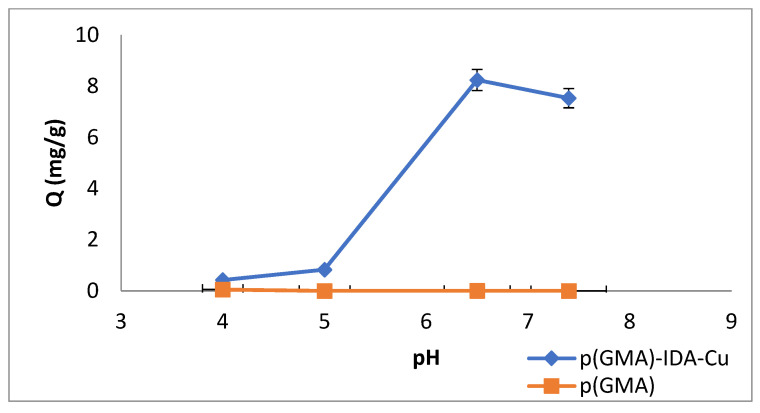
pH Effect (C_Penicillin G-initial_: 0.05 mg/mL, T: 25 °C, t_adsorption_: 2 h).

To investigate the pH effect, the pH values were studied as 4, 5, 6.5, and 7.4. The removal of Pen G with a p(GMA)-IDA-Cu nanoparticle, as shown in Figure 6, was pH-dependent. The Pen G adsorption capacity of the nanoparticle increased as the acidic region moved to the basic region. The maximum Pen G adsorption was in pH 6.5 phosphate buffer.

The pH is an important factor in the affinity interaction system, which is governed by non-covalent interactions. The surface load distribution due to the ionizable groups on the target molecule surface that will lead to pH is a very important factor in the specific affinity interactions with the binding sites in the affinity support material.

In addition, the β-lactam antibiotics, to which the Pen group antibiotics are bound, can be degraded to penicilloic acid, penicilaldehyde, and isopenicillic acid by the protonation of the carboxylic acid groups of the antibiotics in strongly acidic conditions of the nucleophilic β-lactam ring. Degradation is caused by the carbonyl carbon being attacked by nucleophilic agents, making the lactam ring very unstable [6,31]. Therefore, when the pH effect of the nanoparticle on Pen G adsorption was examined, low adsorption values were obtained due to the degradation of the Pen G structure, which was due to the hydrolysis of the β-lactam ring at acidic pH values.

Similarly, in the literature, it has been stated that the optimum pH value for Cu(II) chelate formation is nearly pH = 5.5 [29]. Because the solubility product of the precipitation of Cu(II) is low when the pH value is greater than 5, raising the precipitation problem, the pH values below 5 were used for chelating. This depends on the surface complexes that develop between the chelating ligands of IDA and the Cu(II) ion. The characteristics of the adsorbent surface and the cation’s species distribution also played a role in the metal cation’s ability to bind to the adsorbent. The dominant cation at low pH levels and high Cu(II) ion concentrations was the hydrogen ion. Cu(II) ion adsorption was, therefore, unfavorable at a low pH. The stability of Cu^2+^ chelation also showed an increasing effect for Pen G adsorption [12]. Therefore, this optimum pH value was determined as being optimum for Cu^2+^ chelation and Pen G adsorption.

#### 3.2.2. Initial Concentration Effect

Initial Pen G concentrations of 0.05, 0.1, 0.5, 1, 2, 4, 5, and 10 mg/mL were studied to investigate the effect of initial concentration on the removal of Pen G.

**Figure 7:**
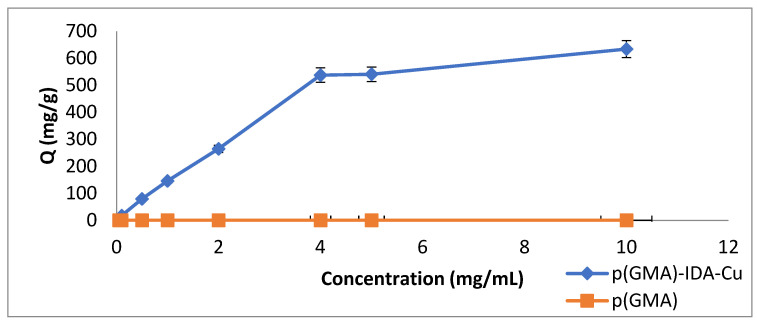
Initial concentration effect (phosphate buffer pH 6.5, T: 25 °C, t_adsorption_: 2 h).

The effect of the Pen G initial concentration on the Pen G adsorption of the p(GMA)-IDA-Cu^2+^ nanoparticle was plotted. As can be seen from Figure 7, by increasing the concentration of Pen G in the solution, the amount of Pen G adsorbed per unit mass to the nanoparticles increased. A high adsorption capacity of 633.92 mg/g was observed at a concentration of 10 mg/mL Pen G. Increasing the initial concentration of Pen G increased the concentration difference (∆C), which is the driving force for adsorption. The Pen G adsorption capacity is also increasing with the increase in the driving force.

In addition, at high values per unit mass of nanoparticle, the 633.92 mg/g Pen G removal was associated with the large surface area of the nanoparticles. In addition, the presence of Cu molecules on the nanoparticles increased the Pen G removal of the p(GMA)-IDA-Cu^2+^ nanoparticle with increasing Pen G concentration.

#### 3.2.3. Ionic Strength Effect

NaCl solution (0.05, 0.1, 0.5, 1, and 2 M) was used at different concentrations to investigate the effect of ionic strength on the removal of Pen G.

**Figure 8:**
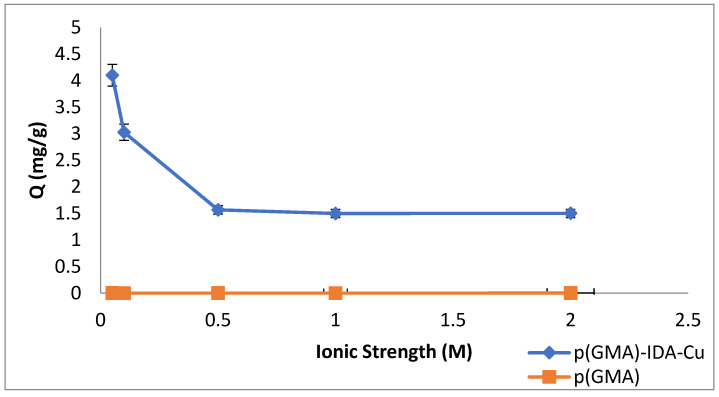
Ionic strength effect (C_Penicillin G-initial_: 0.05 mg/mL; phosphate buffer pH 6.5, T: 25 °C, t_adsorption_: 2 h).

The effect of ionic strength on the Pen G adsorption of p(GMA)-IDA-Cu^2+^ nanoparticles is plotted in Figure 8. To investigate the effect of ionic strength, studies were carried out in five different ionic environments. As can be seen from the graph, the Pen G adsorption of the nanoparticle influenced the adsorption of the nanoparticle significantly in the negative direction in the presence of different ions in the medium. The electrical double layer surrounding the molecules shrank as the ionic strength in the adsorption medium increased. The electrostatic and Van der Waals interactions between the Pen G molecules and the -IDA-Cu(II) complex were affected by ionic strength [29]. A significant reduction in adsorption capacity with salt addition indicated that electrostatic interactions were predominant. Therefore, the interactions between salt ions and Pen G molecules could be to blame for the considerable reduction in the binding capacity of the Pen G caused by the increase in ionic strength.

### 3.3. Time Effect

The time effect on Pen G adsorption was examined with samples taken at 0, 5, 15, 30, 45, 60, 90, and 120 min.

**Figure 9:**
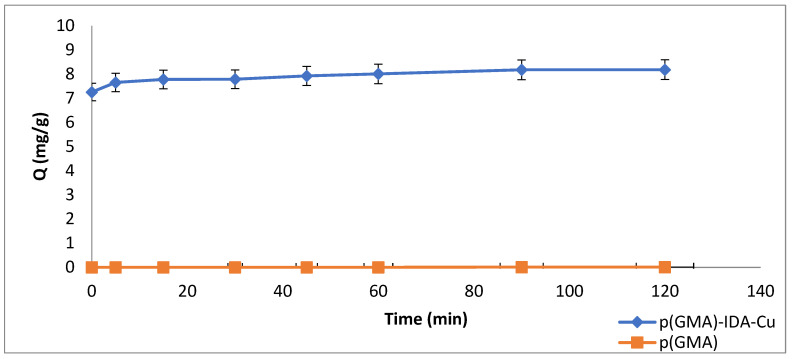
Time effect (C_Penicillin G-initial_: 0.05 mg/mL, pH 6.5 (0.1 M, phosphate buffer), T: 25 °C).

It was observed that the Q (adsorption capacity) values obtained after reaching a certain value reached saturation. As shown in Figure 9, adsorption was stabilized after 15 min, and the maximum adsorption value was reached.

### 3.4. Temperature Effect

**Figure 10:**
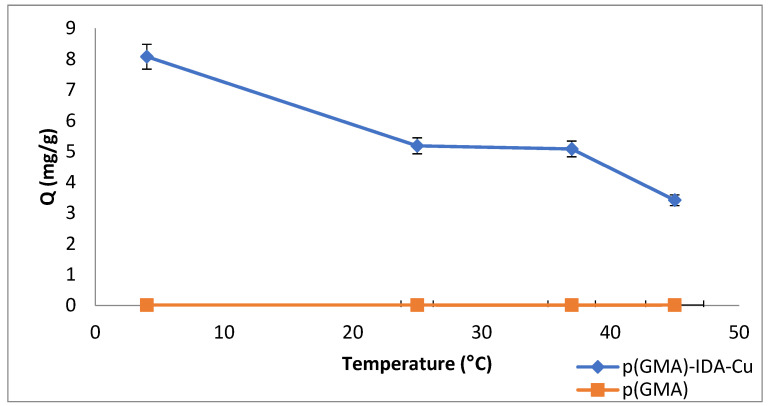
Temperature effect (C_Penicillin G-initial_: 0.05 mg/mL, phosphate buffer pH 7.5, t_adsorption_: 1 h).

The effects of temperature were studied at 4, 25, 37, and 55 °C. The change in adsorption capacity of the p(GMA)-IDA-Cu^2+^ nanoparticle was plotted in Figure 10. The effect of the Pen G adsorption on the temperature was examined at 4–55 °C. It was observed in the obtained graph that the adsorption capacity decreased gradually with increasing temperature values. The Pen G removal was reduced at high temperatures. The lactam ring of beta-lactam antibiotics is chemically very unstable to acid, heat, and beta-lactamase enzymes [31]. The instability of beta-lactam antibiotics at high temperatures has been proven in many articles by experiments [31,32]. Similarly, in this study, it was observed that as the temperature increased, the adsorption capacity decreased due to the deterioration of the Pen G structure.

### 3.5. Reusability Experiment of p(GMA)-IDA-Cu^2+^ Nanoparticles

The adsorption–desorption cycle was repeated five times so that the reproducibility of the synthesized nanoparticles can be determined. The desorption rate was found to be 84.5%.

**Figure 11:**
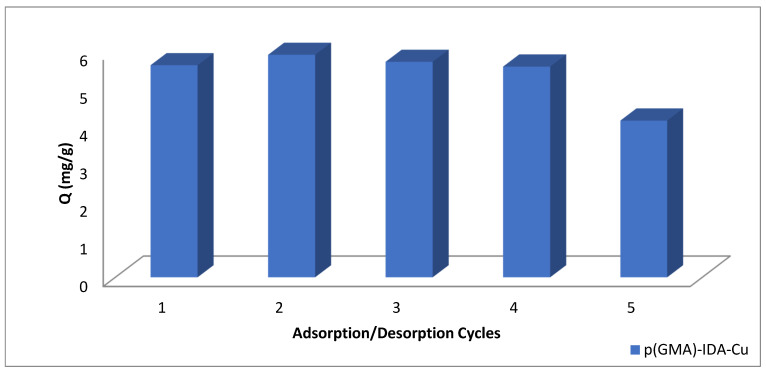
Reusability experiment of p(GMA)-IDA-Cu^2+^ nanoparticles (C_Penicillin G_: 0.05 mg/mL, phosphate buffer pH = 6.5, T: 25 °C, t_adsorption_: 1 h, t_desorption_: 1 h).

As can be seen in Figure 11, there is no significant decrease in adsorption capacity after five repeated adsorption–desorption processes. Cost is also an important factor in the separation of antibiotics, as it is in all separations, and the ability of the synthesized nanoparticle to be used repeatedly with this regenerating ability reduces the cost, while the high adsorption capacity increases the efficiency of the separation of the antibiotics.

### 3.6. Selectivity and Specificity Experiment of Metal-Chelated Nanoparticles

**Figure 12:**
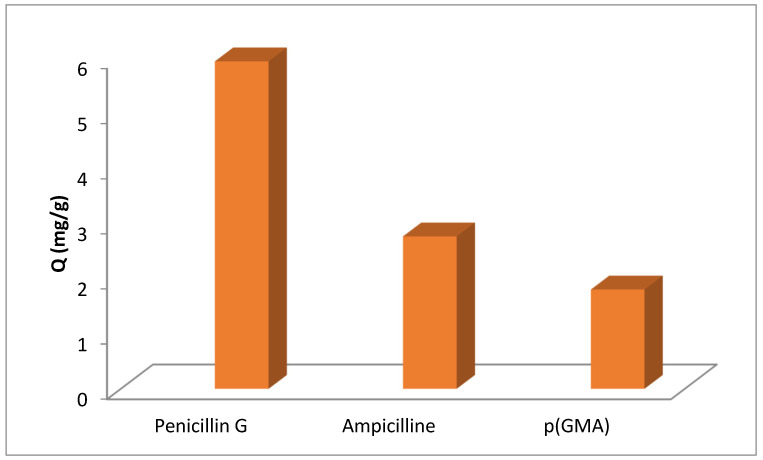
Selectivity and specificity experiment of p(GMA)-IDA-Cu^2+^ nanoparticles (C_Penicillin G_: 0.05 mg/mL, phosphate buffer pH = 6.5, T:25 °C, t_adsorption_: 1 h) and (C_ampicillin_: 0.05 mg/mL, phosphate buffer pH = 6.5, T:25 °C, t_adsorption_: 1 h).

Kd, the distribution coefficients, and K, the selectivity and specificity parameters, for ampicillin, penicillin G, and p(GMA) were examined (Table 1). The selectivity and specificity of the nanopolymer were calculated using Equations (4) and (5). As shown in Figure 12 and Table 1, the Penicillin G adsorption of the p(GMA)-IDA-Cu^2+^ nanopolymer was approximately 2.8-fold more favorable than for the adsorption of ampicillin, another β-lactam antibiotic structurally like Penicillin G, under the same media conditions. In addition, the Penicillin G adsorption of the p(GMA)-IDA-Cu nanopolymer was approximately 3-fold more favorable than for the Pen G adsorption of p(GMA).

## 4. Discussion

Within the scope of this study, nano-sized p(GMA) particles bearing an epoxy group were synthesized. The p(GMA)-IDA nanoparticle was formed by covalently attaching the tridentate immunogenetic acid (IDA) chelator to the accessible reactive epoxy groups on the nanoparticle. Within the scope of the immobilized metal affinity method, nanoparticle immobilization of the interacting metal ion between the carboxyl groups of the IDA used as the ligand and the transition metal copper was achieved. In this method, the coordination complex of the Cu metal ion with the IDA ligand increased the adsorption capacity of the p(GMA)-IDA-Cu^2+^ nanoparticle, which is an immobilized metal affinity adsorbent with selectivity to the target molecule, Pen G.

According to the literature research, various methods and techniques, including microbial inhibition tests, enzyme-linked immunosorbent assays, chromatographic methods, and nanomaterial-based methods, are used for the removal of Pen G from food products and wastewater [6,20,27,31,33,34,35,36,37,38,39,40,41]. All these methods need to be developed to become more precise, which could reduce the analysis times, improve the sample throughput, and reduce the cost per analysis to monitor or remove antibiotics from wastewater and food products [42]. Table 2 presents a comparison of this study with the literature in terms of material size, adsorption capacity, and time.

In this study, Penicillin G removal with the metal-chelated nanoparticles reached its maximum adsorption capacity in a relatively short time (15 min). The adsorption capacity of the nanoparticle reached a high value of 633.92 mg/g. This adsorption capacity of the synthesized metal-chelated nanoparticle was spherical at 150 nm, resulting in a very high specific surface, namely 1072.013 m^2^/g, which must be attributed to its area. In addition, there was no significant decrease in the adsorption capacity of Pen G resulting from the repeated adsorption–desorption process of the p(GMA)-IDA-Cu^2+^ nanoparticle over the five cycles. The nanoparticle had an 84.5% ability to regain its ability to regenerate the product with its regeneration capability, making the widespread use of the system very convenient in terms of reducing the cost, an important factor in removal processes. The penicillin G adsorption of the p(GMA)-IDA-Cu^2+^ nanoparticle was approximately 2.8-fold more favorable than that for the adsorption of ampicillin, another β-lactam antibiotic structurally like Penicillin G, under the same media conditions. In addition, the Penicillin G adsorption of the p(GMA)-IDA-Cu^2+^ nanoparticle was approximately 3-fold more favorable than for the Pen G adsorption of p(GMA).

The metal-chelated nanoparticle system was developed which has nano-size, is environmentally friendly, selective, and highly bioavailable, and has a high adsorption capacity and a relatively low cost. It can be used for the direct removal of Pen G, which is a β-lactam antibiotic widely used as growth factor in animal breeding for antimicrobial treatment from environmental wastewater and food products. The synthesized metal-chelated nanoparticles were presented as a convenient, stable, and inexpensive nanobiotechnological product for the removal of Pen G.

## Figures and Tables

**Table 1:** Selectivity index.

Molecule	p(GMA)-IDA-Cu^2+^		p(GMA)	
	Kd (mL/mg)	K	Kd (mL/mg)	K
Penicillin G	2894.92	-	940.74	3.08
Ampicilline	1045.02	2.77	-	-

**Table 2:** Comparison of this study with the literature.

Used System	Size	Adsorption Capacity	Time	Sample	References
**BLA-imprinted polymers**	25–38 µm particles	9.56 μg/L	NA	Rivers and drinking water	Yin et al., 2010 [31]
**HPLC**	Reverse phase C18 2.5 μm 4.6 × 30 mm XBridge column	7.76 μg/L	10 min	Patient samples from pathology laboratory	Briscoe et al., 2012 [39]
**PenG-imprinted polymers**	25–50 μm	100% cross-reactivity	20 h	Screening from library	Cederfur et al., 2003 [36]
**Nanoscale liquid chromatography (nano-LC) with UV and MS**	0.2 μm pore size	Nano-LC-MS showed 4- to 340-fold reduction in detection limit according to nano-LC-UV	45 min	Drug, milk, and, body fluid samples	Hsieh et al., 2009 [37]
**Dried R. arrhizus and dried activated sludge**	<0.15 mm	1000 mg/L	20–30 min	Aqueous solution	Aksu et al., 2005 [43]
**Molecular-imprinted polymers**	50–100 μm	92 to 103%	20 h	Molecular-engineered assay	Benito-Pena et al., 2006 [27]
**Nanoparticles of titanium dioxide (TiO_2_) doped with Fe^3+^**	Less than 50 nm	90.5%	120 min	Drinking water	Dehghani et al., 2014 [11]
**Hexadecyl Trimethyl Ammonium Bromide modified montmorillonite (HDTMA-Mt)**	NA	88.5 mg/g	60 min	Aqueous solutions	Nourmoradi et al., 2019 [44]
**Molecular-imprinted polymers**	60.38 nm	71.91 g/g	15 min	Laboratory scale	Kuru et al., 2020 [32]
**Molecular-imprinted membrane**	0.570 nm holes	3.50 µg/g	20 min	Tap and natural water	Akbulut Soylemez, 2020 [45]
**PPI@SBA-15/ZIF-8 nanoparticles**	NA	400 mg/g	90 min	Aqueous solutions	Sadeghi et al., 2023 [46]
**This study**	150 nm	633.92 mg/g	15 min	Laboratory scale	

## Data Availability

The data presented in this study are available on request from the corresponding authors.
